# Patient participation and associated factors in the discussions on do-not-attempt-resuscitation and end-of-life disclosure: a retrospective chart review study

**DOI:** 10.1186/s12904-020-00698-8

**Published:** 2021-01-06

**Authors:** Akiko Abe, Masato Kobayashi, Takashi Kohno, Mari Takeuchi, Saori Hashiguchi, Masaru Mimura, Daisuke Fujisawa

**Affiliations:** 1 Department of Neuropsychiatry, Keio University School of Medicine, 35 Shinanomachi, Shinjuku-ku, Tokyo 160-8582 Japan; 2 Palliative Care Center, Keio University Hospital, 35 Shinanomachi, Shinjuku-ku, Tokyo Japan; 3 Keio University School of Medicine, 35 Shinanomachi, Shinjuku-ku, Tokyo Japan; 4 Department of Cardiology, Kyorin University School of Medicine, 6-20-2 Shinkawa, Mitaka-shi, Tokyo Japan; 5 Department of Anesthesiology, Keio University School of Medicine, 35 Shinanomachi, Shinjuku-ku, Tokyo Japan; 6 Division of Patient Safety, Keio University Hospital, 35 Shinanomachi, Shinjuku-ku, Tokyo Japan

**Keywords:** Do-not-attempt-resuscitation (DNAR) orders, Cardiopulmonary resuscitation (CPR), End-of-life discussion, Advance care planning, Patient participation

## Abstract

**Background:**

Patient participation is a key foundation of advance care planning (ACP). However, a patient himself/herself may be left out from sensitive conversations such as end-of-life (EOL) care discussions. The objectives of this study were to investigate patients’ participation rate in the discussion of Cardiopulmonary Resuscitation (CPR) / Do-Not-Attempt-Resuscitation (DNAR) order, and in the discussion that the patient is at his/her EOL stage (EOL disclosure), and to explore their associated factors.

**Methods:**

This is a retrospective chart review study. The participants were all the patients who were hospitalized and died in a university-affiliated teaching hospital (tertiary medical facility) in central Tokyo, Japan during the period from April 2018 to March 2019. The following patients were excluded: (1) cardiopulmonary arrest on arrival; (2) stillbirth; (3) under 18 years old at the time of death; and (4) refusal by their bereaved family. Presence or absence of CPR/DNAR discussion and EOL disclosure, patients’ involvement in those discussions, and their associated factors were investigated.

**Results:**

CPR/DNAR discussions were observed in 336 out of the 358 patients (93.9%). However, 224 of these discussions were carried out without a patient (patient participation rate 33.3%). Male gender (odds ratio (OR) = 2.37 [95% confidence interval (CI) 1.32–4.25]), living alone (OR = 2.51 [1.34–4.71]), and 1 year or more from the date of diagnosis (OR = 1.78 [1.03–3.10]) were associated with higher patient’s participation in CPR/DNAR discussions. The EOL disclosure was observed in 341 out of the 358 patients (95.3%). However, 170 of the discussions were carried out without the patient (patient participation rate 50.1%). Patients who died of cancer (OR = 2.41[1.45–4.03]) and patients without mental illness (OR=2.41 [1.11–5.25]) were more likely to participate in EOL disclosure.

**Conclusions:**

In this clinical sample, only up to half of the patients participated in CPR/DNAR discussions and EOL disclosure. Female, living with family, a shorter period from the diagnosis, non-cancer, and mental illness presence are risk factors for lack of patients’ participation in CPR/DNAR or EOL discussions. Further attempts to facilitate patients’ participation, based on their preference, are warranted.

**Supplementary Information:**

The online version contains supplementary material available at 10.1186/s12904-020-00698-8.

## Background

Advanced care planning (ACP) is defined as a process of assessment and person-centered dialogue to establish an individual’s needs and goals of care [1], enabling individuals to define their goals and preferences for future medical treatment [1, 2]. ACP can improve patient-clinician communication quality, reduce unwanted admission to hospitals, increase the use of palliative care, and increase patient satisfaction and quality of life [3].

Providing patients with appropriate information regarding the expected course of illness and prognosis [4, 5] and discussing their medical care preferences with them are essential parts of ACP [5]. ACP could be done in the provision of care to people at various stages of their illness, but its content can be more targeted as their health condition worsens [1]. During these discussions on ACP, the topic of patient preference of whether and to what extent to receive life-sustaining interventions at their EOL stage often comes up (e.g. cardiopulmonary resuscitation (CPR) and Do-Not-Attempt-Resuscitation (DNAR) orders). Having EOL discussion with patients early in the course of their illnesses can result in higher concordance between patients’ prior-stated wishes and actually-received treatments, decrease aggressive care at the EOL, and lead to a better quality of EOL care [6].

The ways how ACP is implemented are influenced by many factors, such as cultural backgrounds, medical systems, legal frameworks, patients’ sociodemographic and clinical characteristics, and the preference of patients and their families [2]. Respect for autonomy is an important value in medical ethics, and the involvement of patients themselves is an essential part of ACP. However, the level of patients’ involvement varies between different societies and clinical settings. Especially on sensitive issues such as CPR/DNAR orders and EOL disclosure, a patient himself/herself is sometimes left out from the discussion [7–9].

Only a few studies have evaluated the rate of patients’ participation in such discussions. The concerned studies have been limited to those involving a specific type of illness (e.g. cancer and heart failure) or limited to specific treatment settings (e.g. in palliative care units) [7–13]. Also, factors that associate with patient involvement in such discussions have not yet been clarified.

Therefore, the current study aimed to investigate patients’ participation rates in the discussions on CPR/DNAR orders and EOL disclosure among the patients who died in a hospital due to any cause of illness. The factors associated with the participation of patients themselves in the discussion were also explored.

## Methods

### Cohort description

This study, a retrospective chart review, was conducted at Keio University Hospital, a university-affiliated teaching hospital (tertiary medical facility) in central Tokyo, Japan. All the patients who were hospitalized and died in the study site during the period from April 2018 to March 2019 were eligible. The following patients were excluded: (1) cardiopulmonary arrest on arrival (CPAOA); (2) stillbirth; (3) under 18 years old at the time of death; and (4) refusal by their bereaved family.

### Outcome measures

The co-primary outcomes were participation of a patient himself/herself in (1) the discussion on CPR/DNAR and (2) the discussion where a clinician disclosed that the patient is at his/her end-of-life stage (EOL disclosure). These information, with related patients’ characteristics, were obtained from the medical chart. The EOL disclosure was defined as a discussion where a treating clinician informed that the patient’s death was approaching in a short period of time (within weeks or months), when aggressive life-prolonging treatment was not considered useful. Initially, two physicians (AA and DF) reviewed patients’ medical charts independently and identified presence/absence of concerned documentation. When the results between the two physicians were not concordant, discussion was held until an agreement was reached.

### Discussions on CPR/DNAR

We examined the discussions on preferences for CPR (mechanical ventilation and chest compression) and DNAR in the event of a cardiopulmonary arrest with a low probability of recovery. The following information was obtained; (i) the presence/absence of the discussion on CPR/DNAR, (ii) the date of the discussions on CPR/DNAR (number of days before death), (iii) the participants in the discussion (patient, family, and medical staff), (iv) their preference for CPR or DNAR, and (v) the reason why the patient did not participate in the discussion (when the patient was absent from the discussion). The cases where the patients did not clearly state their intentions about participation in the discussion or where their family’s wishes were not clear in the medical records were defined as “doctors’ judgment”.

### EOL disclosure

We defined EOL disclosures as the prognostic announcements by treating physicians to patients and/or their families that the patient was at their end-of-life stage. The following information was obtained; (i) the presence/absence of an EOL disclosure, (ii) the date of the EOL disclosure, (iii) the participants in the EOL disclosure; (iv) whether there was a chance for the patient to participate in the discussion on a later date (if the patient did not participate in the first discussion).

### Patients’ characteristics

The patients’ sex, age, marital status, family structure, cause of death, length of time since the diagnosis to death, length of the last hospital stay, number of hospitalizations in the last 2 years, and history of mental illness were collected. The following conditions were defined as mental illness: schizophrenia, mood disorders, neurosis, dementia, epilepsy, mental retardation, pervasive developmental disorders, substance abuse, and continuous use of psychotropic drugs. Delirium was not regarded as a mental illness.

### Statistical analysis

Since this is an explanatory study, we did not set a target sample size. After performing the descriptive analysis, the participants were divided into two groups (based on whether or not a patient participated in CPR/DNAR discussions and EOL disclosures). The characteristics of the patients were compared between these groups. Categorical variables and continuous variables were compared using Chi-square tests and non-parametric tests, respectively. Multivariate logistic regression analyses were conducted to explore factors that associate with patients’ participation in the discussions. We chose the following independent factors; 1) background: age, sex, and family structure, 2) medical factors: diagnosis (cancer or non-cancer), presence/absence of mental illness, number of hospitalization, and the period from the diagnosis to the death. We used continuous variables for “age” and “number of hospitalization”. We dichotomized “period from the diagnosis to the death” into “more than 1 year” or “less than 1 year”.

A *p*-value of < 0.05 was considered statistically significant. All analyses were conducted using the IBM SPSS version 24.0 and 25.0 (IBM Corp., Armonk, NY, USA).

### Ethical consideration

This study complied with the Declaration of Helsinki and was approved by the ethics committee of Keio University Hospital (Approval number: 20190034). Permission to collect and analyze data was given by the bereaved family by opt-outs.

## Results

### Sample characteristics

Of the 377 hospitalized patients who died during the study period, 19 patients were excluded (CPAOA: *n*=12, stillbirth: *n*=2, under 18 years old: *n*=5, and refusal by their bereaved family: *n*=0). Finally, the data of 358 patients were subjected to analyses (Supplemental material 1).

The patient characteristics are shown in Table 1. Two-thirds of the deceased patients were male, and the patients’ mean age was 70 years old. Approximately 60% of the patients died of cancer.

**Table 1:** Demographic and clinical characteristics of the patients (*n*=358)

Characteristics	n (%)
Age, years (mean, SD)	69.9±15.1
Gender: Male	229 (64.0%)
Marital status: Married	236 (65.9%)
Family structure	
Living alone	67 (18.7%)
Couple only	134 (37.4%)
Living with other families	157 (43.9%)
Number of hospitalization in the last two years (mean, SD)	3.5±3.0
Diagnosis	
Cancer	206 (57.5%)
Lung	47 (13.1%)
Lymphoma	18 (5.0%)
Colorectal	15 (4.2%)
Gastric	12 (3.4%)
Uterine	11 (3.1%)
Renal	10 (2.8%)
Gallbladder, bile duct	9 (2.5%)
Pancreatic	9 (2.5%)
Leukemia	9 (2.5%)
Others	66 (18.4%)
Non-cancer	152 (42.5%)
Respiratory disease	54 (15.1%)
Cardiovascular disease	29 (8.1%)
Liver disease	16 (4.5%)
Cerebrovascular disease	15 (4.2%)
Others	38 (10.6%)
Presence of mental illness: yes	51 (14.2%)

*SD* standard deviation

### Discussions on CPR/DNAR

**Fig. 1:**
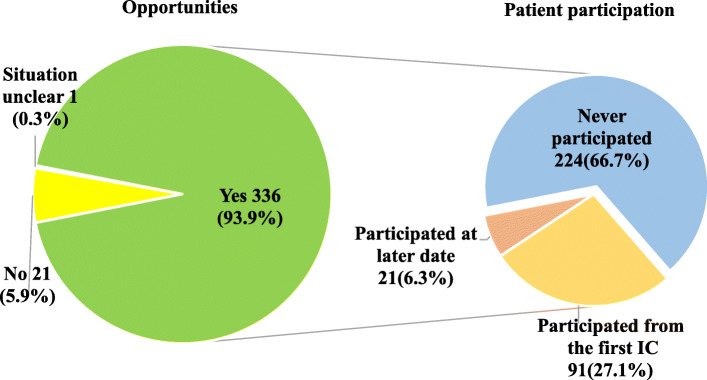
Prevalence and participation rate of CPR/DNAR discussions. CPR: cardiopulmonary resucitation, DNAR: do not attempt resucitation, IC: informed consent

Discussions on CPR/DNAR were observed in 336 out of the 358 patients (93.9%). Twenty-one patients and their families had no opportunity to discuss CPR/DNAR due to unexpected death. In one patient, there was no written information on CPR/DNAR discussions (Fig. 1). For 305 patients (90.8%), the discussions took place while the patients were in the hospital (hospitalized) and for 31 patients (9.2%), during an outpatient visit.

Of the 336 patients who had CPR/DNAR discussions, 112 patients (33.3%) participated in the discussions. However, 224 of the discussions (66.7%) were carried out without the patient. Among 112 patients, 91 patients (81.3%) participated in the first discussion, and the rest participated at later opportunities (after discussions were first held between the family and the physician in charge). The most common reason for patients’ not participating in the first discussion was the patient’s decreased consciousness (*n*=119: 48.6%), followed by doctors’ judgment (*n*=107: 43.7%), and requests by their family (*n*=11: 4.5%). In those first discussions, approximately 60% of the patients requested DNAR orders (with patient: 63.7%, without patient: 57.1%), 10% requested full-code CPRs (with patient: 9.9%, without patient: 13.1%), while 30% undecided (with patient: 26.4%, without patient: 29.8%). The presence or absence of the patient in the discussions did not have a significant influence on the decision. When the patient was absence from the DNAR discussion, DNAR decisions were made by patients’ family as a result of discussion between family members and physician. Eventually, DNAR decisions were made in 96.4% of the cases.

The characteristics of the patients who participated in the CPR/DNAR discussion are shown in Table 2. The patients who were male, younger, living alone, without mental illness, with a larger number of hospital admissions in the last 2 years, with a longer period from diagnosis of the main disease until death, and who died of cancer were more likely to participate in CPR/DNAR discussions. Also, the patients were more likely to participate in the discussions when the discussions took place before the patient’s last admission to the hospital.

**Table 2:** Characteristics of patients who participated or did not participate in CPR/DNAR discussion and EOL disclosure

	Participation in CPR/DNAR discussion			Participation in EOL disclosure		
	Yes (*n*=112)	No (*n*=224)	*p* value	Yes (*n*=171)	No (*n*=170)	*p* value
Gender						
Male	83 (74.1%)	135 (60.3%)	0.012	117 (68.4%)	104 (61.2%)	0.16
Female	29 (25.9%)	89 (39.7%)		54 (31.6%)	66 (38.8%)	
Age, years (mean, SD)	68.1±13.5	70.7±15.7	0.034	67.4±13.5	72.4±15.7	<0.001
Marital status						
Married	69 (61.6%)	151 (67.4%)	0.29	118 (69.0%)	108 (63.5%)	0.29
Unmarried	43 (38.4%)	73 (32.6%)		53 (31.0%)	62 (36.5%)	
Family structure						
Living alone	32 (28.6%)	32 (14.3%)	0.002	40 (23.4%)	25 (14.7%)	0.041
Living with family	80 (71.4%)	192 (85.7%)		131 (76.6%)	145 (85.3%)	
Presence of mental illness						
Yes	9 (8.0%)	37 (16.5%)	0.033	14 (8.2%)	32 (18.8%)	0.004
No	103 (92.0%)	187 (83.5%)		157 (91.8%)	138 (81.2%)	
Diagnosis						
Cancer	75 (67.0%)	121 (54.0%)	0.023	122 (71.3%)	77 (45.3%)	<0.001
Non-cancer	37 (33.0%)	103 (46.0%)		49 (28.7%)	93 (54.7%)	
Number of hospitalization in the last two years (mean, SD)	3.7±2.8	3.5±3.2	0.049	4.0±3.2	3.2±2.9	<0.001
Period from the diagnosis to the death, days (mean, SD)	1103±1203	998±1905	0.006	1261±2120	815±1299	0.001
Length of the last hospital stay, days (mean, SD)	32.1±38.4	35.9±44.3	0.46	32.9±38.2	35.8±46.0	0.66
Period from the first applicable IC to the death, days (mean, SD)	100.8±239.0	66.2±170.5	<0.001	113.8±240.5	35.7±72.6	<0.001
Place of discussion						
Outpatient service	14 (12.5%)	17 (7.6%)	0.143^a^	37 (21.6%)	18 (10.6%)	0.006^a^
Inpatient service	98 (87.5%)	207 (92.4%)		134 (78.4%)	152 (89.4%)	
last hospitalization	67 (59.8%)	178 (79.5%)	<0.001^b^	89 (52.0%)	134 (78.8%)	<0.001^b^
other hospitalizations	31 (27.7%)	29 (12.9%)		45 (26.3%)	18 (10.6%)	

*CPR* cardiopulmonary resuscitation, *DNAR* Do-Not-Attempt-Resuscitation, *EOL* End Of Life, *IC* informed consent, *SD* standard deviation

^a^outpatient vs. inpatient (both last hospitalization and others)

^b^last hospitalization vs. other hospitalizations

The logistic regression analysis using these factors as independent variables demonstrated that male gender (odds ratio (OR) = 2.37 [95% confidence interval (CI): 1.32–4.25]), living alone (OR = 2.51 [1.34–4.71]) and 1 year or more from diagnosis (OR = 1.78 [1.03–3.10]) were associated with the participation of patients themselves in the CPR/DNAR discussions (Table 3).

**Table 3:** Odds ratios for participation in CPR/DNAR discussions

Variable	Odds ratio	95%-CI	*p*-value
Age	0.99	0.97–1.01	0.30
Male gender	2.37	1.32–4.25	< 0.01
Number of hospitalization	0.99	0.91–1.07	0.78
Living alone	2.51	1.34–4.71	< 0.01
Cancer (vs. non-cancer)	1.72	0.98–3.01	0.06
Absence of mental illness	2.16	0.87–5.35	0.095
More than one year from the diagnosis (vs. less than one year)	1.78	1.03–3.10	0.04

*CI* confidence interval

### EOL disclosure

**Fig. 2:**
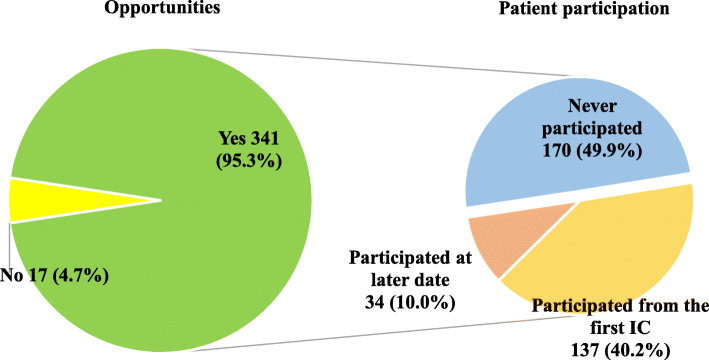
Prevalence and participation rate of the EOL disclosure. EOL: end of life, IC: informed consent

EOL disclosures were observed in 341 out of the 358 patients (95.3%). Seventeen patients and their families had no opportunity to participate in EOL disclosure because they died unexpectedly due to a sudden change in their condition (Fig. 2). For 286 patients (83.9%), the discussions took place when the patients were in the hospital (hospitalized) and for 55 patients (16.1%), during an outpatient visit.

Of the 341 patients who had an EOL disclosure, 171 patients (50.1%) participated in the discussions. However, 170 of the discussions (49.9%) were carried out without a patient. Of the 171 patients, 137 patients (81.1%) participated from the first opportunity and the rest participated at later opportunities (after a discussion was first held between the family and the physician in charge). The most common reason for patients’ not participating at the first opportunity was decreased consciousness (*n*=108: 52.9%), followed by doctors’ judgment (*n*=83: 40.7%) and by families’ requests (*n*=8: 3.9%).

The characteristics of the patients who participated in the EOL disclosure are shown in Table 2. The patients who were younger, living alone, without mental illness, with a larger number of hospital admissions in the last 2 years, with a longer period from diagnosis of the main disease until death, and who had a diagnosis of cancer were more likely to participate in the EOL disclosure. Also, the patients were more likely to participate in the discussions when the disclosure took place at outpatient service or when the disclosure took place before the patient’s last admission to the hospital.

The logistic regression analysis demonstrated that dying of cancer (OR=2.41[1.45–4.03]) and without mental illness (OR=2.41 [1.11–5.25]) are associated with higher patients’ participation in the EOL disclosure (Table 4).

**Table 4:** Odds ratio for participation in the EOL disclosure

Variable	Odds ratio	95%-CI	*p*-value
Age	0.99	0.97–1.00	0.09
Male gender	1.36	0.81–2.28	0.25
Number of hospitalization	1.06	0.97–1.15	0.22
Living alone	1.83	0.97–3.47	0.06
Cancer (vs. non-cancer)	2.41	1.45–4.03	< 0.01
Absence of mental illness	2.41	1.11–5.25	0.026
More than one year from the diagnosis (vs. less than one year)	1.66	0.99–2.77	0.054

*CI* confidence interval

### Association between the participation in CPR/DNAR discussions and the EOL disclosure

There was a significant association between the patients’ participation in CPR/DNAR discussions and the patients’ participation in the EOL disclosure (Spearman’s rank correlation coefficient = 0.49, *p* < 0.05).

## Discussion

The current study revealed that the patient participation rate of CPR/DNAR discussions was 33.3%. Male gender, living alone, and 1 year or more from the diagnosis were associated with a higher patient’s participation in CPR/DNAR discussions. The patient participation rate in the EOL disclosure was 50.1%. Patients who died of cancer and patients without mental illness were also more likely to participate in the EOL disclosure.

Approximately in 5% of deceased patients, EOL discussion (including CPR/DNAR discussion) did not take place at all. This was mostly due to unexpected in-hospital death. Considering that unexpected in-hospital cardiac arrest occurs in a nonnegligible proportion of hospitalized patients [14], all patients who were admitted to the hospital could be eligible for discussions on EOL care in preparation for deterioration. Indeed, the American Heart Association Guidelines for Cardiopulmonary Resuscitation and Emergency Cardiovascular Care recommended that physicians should initiate discussions on CPR/DNAR with all the patients admitted for medical and surgical care [15].

Only one-third of our participants had a chance to participate in their CPR/DNAR discussions, and only a half had a chance to participate in the EOL disclosure. These participation rates are higher than those in the previous studies in Japan, which ranged from 0 to 6% [10, 12, 13], but lower than in those of the studies from USA, Switzerland and Finland, which ranged from 37 to 80% [7–9, 16–19]. The definitions of EOL discussions varied among the studies and a simple comparison was difficult, however, there could be several possible reasons. First, the rate of patients’ participation increases as the time of the survey becomes more recent [10–13]. In Japan, the Ministry of Health, Labor and Welfare first published guidelines on the decision-making process for end-of-life care in 2007 (which was revised in 2018). They stipulate that the will of the patient is most important and needs to be ensured. When the patient’s will is not certain, their family is supposed to serve as the patient’s proxy and have well-informed discussion with medical professionals. The rise in the participation rate probably reflects these growing interest in ACP. Second, the participation rate can be influenced by the legal background and health care system of the patient’s society. Patient involvement ought to be higher in societies where ACP and/or advance directives are mandatory in a certain situations (e.g., USA [20] and Taiwan [21]). Also, cultural perspectives can be influential. Japanese culture places value in ambiguity rather than explicitness, compared with Western societies. For example, a nation-wide research on the concept of “good death” indicated that many Japanese do not want to know the seriousness of their medical conditions [22]. The majority of Japanese general population considered “dying without awareness that one is dying” as an important factor to achieve a good death, and approximately half of them considered that “not being informed of bad news” was an important issue during the last days of life. Only 50–69% of Japanese participants agreed to such concept that “knowing what to expect about one’s physical condition” helps them achieve a “good death”, while 96% of the USA participants agreed to such conception [23].

Decreased consciousness hampered approximately one third of the patients from participation in the CPR/DNAR discussions. These discussions should have been initiated earlier for these patients. In principle, it is better to start the ACP earlier, especially in patients who have life-threatening diseases [2, 4]. In fact, in the current study, the patients were more likely to participate in the discussion when the discussion took place during an outpatient visit or before the patient’s last admission to the hospital. The chance of patient involvement seemed to increase if the discussions were carried out earlier. However, the actual timing of the ACP is influenced by many factors such as patient’s preferences, readiness, and medical condition [1, 2, 15]. Using routine assessments, such as the Advance Care Planning Readiness Scale (ACPRS) [24], may help promote ACP. A systematic intervention comprising training of clinicians based on a manual (the Serious Illness Conversation Guide), family materials, and system changes (patient identification using the “surprise question”, email reminders, and documentation templates on ACP) has resulted in an improved implementation of ACP [25].

Approximately 40% of the patients did not participate in their first discussion due to the judgment of their treating physicians. According to an international study that surveyed physicians’ attitudes toward patient autonomy, 82% of the Japanese palliative-care physicians agreed that patients should be informed first of their serious medical condition [26], however, the current study found that, in reality, physicians tended to talk to the family first.

Medical professionals need to have sufficient skills to talk about diagnosis, prognosis, death, and dying with individuals and their families [1]. Providing physicians with appropriate educational opportunities and training, such as end-of-life care, psychological support for patients and their families, and communication skills training is essential [21].

The current study elucidated the factors that relate to patients’ participation in discussions on CPR/DNAR and the EOL disclosure. Patient involvement in the discussions on CPR/DNAR was influenced mainly by sociodemographic factors rather than medical factors.

Male patients were more likely to be involved in discussions on CPR/DNAR. There have been only a few studies that have examined the association between gender and patients’ involvement in CPR/DNAR discussions. In a multisite registry study in the United States, Perman et al. reported that women are more likely than men to establish DNAR instructions [27]. Other studies, including a study that enrolled hospitalized older adults who required a surrogate decision-maker in the United States [16] and a Taiwanese study that enrolled cancer patients [28], demonstrated no significant gender difference. We speculated that there may be paternalistic perspectives in Japan where autonomy is emphasized more among men than women, while women need to be “protected” from serious medical information. In addition, gender distribution of our sample, which was male-dominant, was different from that of general population in Japan (49% are male) [29]. The reason that male patients who died in our hospital comprised the larger proportion is unknown. Male patients may be somehow less likely to be transferred to other facilities or less likely to be discharged during their end-of-life period. Further multisite studies are needed to uncover potential mechanisms of gender difference.

The results of our study also indicated that patients who lived alone were more likely to participate in CPR/DNAR discussions; probably due to the practical reason that patients living alone lack clear proxy decision-makers.

Furthermore, the patients who had 1 year or more to live from the date of diagnosis, were more likely to participate in CPR/DNAR discussions. This result was consistent with a previous Japanese study [30]. The longer the course of the illnesses is, the more prepared the patients become for the future (including death). Trust between the patient and their treating physician may be cultivated during the course of the illness. The treating physicians have more opportunities to understand the patient’s background, personality, and sense of value, which makes sensitive discussion with the patient easier.

The patients who died of cancer were more likely to participate in the EOL disclosure than patients who died of non-cancer illnesses. This is probably because the course of non-cancer diseases, such as heart failure and chronic respiratory diseases are generally less predictable than that of cancer [31]. However, clinical practice such as the use of the “surprise question” - a simple question for clinicians to ask themselves “Would I be surprised if this patient died in the next 12 months?” - has been shown to help identifying patients at high risk of death in the short term [32] in samples of patients with cancer [33], decompensated heart failure [34], and end-stage chronic kidney disease [35].

Patients with mental illness were less likely to participate in EOL disclosure. The mental conditions in the current study were roughly classified into the following three categories; psychological distress (depression and anxiety), serious mental illnesses (schizophrenia, bipolar disorder, and psychotic depression), and cognitive disorders (dementia and intellectual disorders). Several studies showed that an accurate understanding of the prognosis was associated with elevated depression and anxiety [36, 37], thus it is well-understandable that clinicians feel afraid that telling their patients that they are at the EOL stage may worsen their mental conditions. However, since EOL discussions are associated with less aggressive medical care near death and early hospice referrals [9] and most patients with metastatic cancer want detailed prognostic information [38], disclosing accurate prognostic information while minimizing the psychological distress of patients is a challenging but critical issue [39]. Clinicians may consider that patients with serious mental illnesses or cognitive disorders lack decision-making capacity and are not eligible for EOL discussions, which is not necessarily correct. Clinicians should try their best to let the patient be involved in decision making while at the same time considering patients’ mental capacities [40].

The current study has a few limitations. First, since this was a retrospective chart review study, some potential factors that may influence patients’ participation in CPR/DNAR discussions and the EOL disclosure, such as the patient’s decision-making capacity, were not examined. Second, since this was a single-center study with moderate sample size, the generalizability of the results is limited. Gender distribution of our sample, which was male-dominant, was different from that of general population. The study site was an urban acute-care hospital without a palliative care unit, and a substantial proportion of patients were transferred to another hospital or to a home-based hospice program at their EOL, where the discussions on CPR/DNAR and EOL care were likely to have occurred. Third, the quality of the chart documentation may have differed depending on the doctor who wrote it. Also, undocumented covert discussions between clinicians and patients were not detectable.

## Conclusions

Despite these limitations, our study provided a real-world clinical picture of the practice of CPR/DNAR and EOL discussions in patients with various diseases. To the best of authors’ knowledge, this is the first study to elucidate the factors that relate to patients’ participation in the discussions on CPR/DNAR and the EOL disclosure. In this clinical sample, patients’ participation in CPR/DNAR discussions and the EOL disclosure was modest. Female, living with family, a shorter period from the diagnosis, non-cancer, and mental illness presence are risk factors for lack of patients’ participation in CPR/DNAR or EOL discussions. Further attempts to facilitate patients’ participation, based on their preference, are warranted.

## Supplementary Information

**Additional file 1.** Flow diagram.

## Data Availability

Our IRB does not permit the data to be shared publicly. Raw data may be obtained from the corresponding author on reasonable request.

## Abbreviations

ACP: Advance care planning
CPAOA: Cardiopulmonary arrest on arrival
CPR: Cardiopulmonary Resuscitation
DNAR: Do-Not-Attempt-Resuscitation
EOL: End-of-life
