# Supplementary material for: Patient participation and associated factors in the discussions on do-not-attempt-resuscitation and end-of-life disclosure: a retrospective chart review study
Source: BMC Palliat Care. 2021 Jan 6;20:6. doi: 10.1186/s12904-020-00698-8 (PMC7789264; doi:10.1186/s12904-020-00698-8)
Supplement: Supplementary file 1 — Additional file 1. Flow diagram. [file 12904_2020_698_MOESM1_ESM.docx]

Supplemental material 1. Flow diagram

Hospitalized and died during the study period (n=377)

Excluded (n=19)

(1) Cardiopulmonary arrest on arrival (CPAOA) (n=12)

(2) Stillbirth (n=2)

(3) Under 18 years old at the time of death (n=5)

(4) Refusal by their bereaved family (n=0)

Subjected to analyses (n=358)
